# Rewiring of the Apoptotic Rheostat in HTLV-1 Infection and Adult T-Cell Leukemia/Lymphoma

**DOI:** 10.3390/cancers18152494

**Published:** 2026-08-04

**Authors:** Arezoo Darbandi, Vittoria Raimondi, Francesco Ciccarese, Donna M. D’Agostino, Vincenzo Ciminale

**Affiliations:** 1 Veneto Institute of Oncology IOV-IRCCS, 35128 Padova, Italy; arezoo.darbandi@iov.veneto.it (A.D.); vittoria.raimondi@iov.veneto.it (V.R.); francesco.ciccarese@iov.veneto.it (F.C.); 2 Department of Biomedical Sciences, University of Padova, 35131 Padova, Italy; 3 Department of Surgery, Oncology and Gastroenterology, University of Padova, 35128 Padova, Italy

**Keywords:** HTLV-1, apoptosis, BCL-2

## Abstract

Human T-cell leukemia virus type 1 (HTLV-1) is the etiological agent of an aggressive malignancy of mature T-cells termed adult T-cell leukemia/lymphoma (ATLL), as well as a spectrum of chronic inflammatory diseases. A defining feature of HTLV-1 infection is a profound dysregulation of apoptotic pathways, which is a key determinant of long-term viral persistence and favors malignant transformation. This review describes the mechanisms through which HTLV-1 gene products, including Tax and HBZ, reprogram host-cell signaling controlling the “apoptotic rheostat”, an integrated network connecting redox metabolism, and response to apoptotic cues and immune pressure. We also highlight emerging therapeutic strategies to restore sensitivity to apoptosis, including BH3-mimetic drugs and rational combination approaches. Deciphering how HTLV-1 reconfigures the apoptotic network provides a conceptual and therapeutic framework for targeting death pathway vulnerabilities in ATLL and potentially other hematological neoplasms.

## Simple Summary

Human T-cell leukemia virus type 1 (HTLV-1) causes an aggressive blood cancer termed adult T-cell leukemia/lymphoma (ATLL), as well as chronic inflammatory diseases. One important way HTLV-1 persists in the infected host is by interfering with programmed cell death (apoptosis), a natural process that removes damaged or unwanted cells. By blocking apoptosis, the virus promotes the survival of infected cells and increases the risk of leukemia development. This review explains how HTLV-1 changes key survival and death signals inside infected cells. It also discusses treatment approaches targeting these mechanisms to restore cancer cells’ sensitivity to apoptosis with the aim of improving the clinical outcome of patients with ATLL.

## 1. Introduction

Human T-cell leukemia virus type 1 (HTLV-1) (also referred to as human T-lymphotropic virus 1) was the first human retrovirus to be identified [1] and is the only human retrovirus with a direct etiological link to cancer [2,3]. HTLV-1 infects an estimated 10 million people worldwide, with endemic regions in Japan, sub-Saharan Africa, the Caribbean basin, South America, Melanesia, and Australia [4,5]. The infection is transmitted through breastfeeding, sexual intercourse, blood transfusion, and needle sharing, mainly through transfer of virus-producing lymphocytes rather than free virus particles [6].

HTLV-1 is the etiologic agent of two distinct, severe diseases—an aggressive T-cell malignancy termed Adult T-cell Leukemia/Lymphoma (ATLL) [7] and a progressive demyelinating neurological disease termed Tropical Spastic Paraparesis/HTLV-Associated Myelopathy (TSP/HAM) [8]. ATLL and TSP/HAM arise after years to decades of clinical latency, with proviral load, gender, MHC class I haplotype, and other genetic factors linked to disease development. The lifetime risk of an HTLV-1-infected person developing ATLL is about 2–6%, while risk estimates for TSP/HAM range from about 0.25% to nearly 4% depending on the population under study [8,9]. Other pathologies linked to HTLV-1 infection include HTLV-1-associated uveitis, infective dermatitis, rheumatic and autoimmune disorders, opportunistic infections, and lung pathologies [10,11]. On the whole, HTLV-1 infection is associated with an increased risk of premature death [12].

HTLV-1 is a member of the *Deltaretrovirus* genus in the *Orthoretrovirinae* subfamily of retroviruses [13]. A hallmark of all retroviruses is their persistence in the host after integration of the viral genome into the host cell genome. After establishing infection in a new host, HTLV-1 persists and expands mainly through “mitotic spread” of cells containing the proviral genome and, to a minor extent, through virions infecting new cells [6,14].

HTLV-1 is mainly detected in CD4^+^ T-cells and less frequently in CD8^+^ T-cells in infected individuals; it can also infect other cell types, including dendritic cells, monocytes, and macrophages [6]. Interactions between viral proteins and the host cell lead to a complex rewiring of cell signaling pathways that drive proliferation, support survival, and promote genome instability, resulting in clonal expansion of infected T-cells [15]. Neoplastic transformation driven by HTLV-1 is thus tightly linked to the viral replication strategy based on mitotic spread.

ATLL was first described in 1977 as an aggressive mature T-cell neoplasm affecting adults in Japan, most of whom originated from the Kyushu region [16]. ATLL is classified into four clinical subtypes termed acute, lymphoma, chronic, and smoldering, with the acute form being most prevalent [17]. In all of the subtypes, antibodies to viral proteins are present, and malignant cells show monoclonal integration of the provirus. Additional clinical features vary among the subtypes and individual patients and include generalized lymphadenopathy, hepatosplenomegaly, cutaneous lesions, elevated LDH, hypercalcemia, and involvement of the bone marrow, gastrointestinal tract, and central nervous system [17]. ATLL cells from most patients are CD3^+^/CD4^+^/CD25^+^/CCR4^+^ and exhibit marked resistance to chemotherapy [18]; even aggressive multi-agent regimens typically produce only transient responses, resulting in a median survival of less than one year for patients with acute or lymphoma ATLL [18].

HTLV-1 is studied in a variety of in vitro cell models such as neoplastic cells from patients with ATLL, cell lines stabilized from lymphocytes of patients with ATLL or TSP/HAM, in vitro-transformed T cell lines generated by co-cultivating normal PBMC or umbilical cord blood cells with patients’ cells, and cell lines transfected with HTLV-1 molecular clones or with vectors expressing individual viral proteins. Animal models to study HTLV-1 infection and pathogenesis include mice, rabbits, rats, non-human primates, and, most recently, ‘humanized’ immunodeficient mice reconstituted with a normal human hematopoietic system [19]. Information on HTLV-1 pathobiology has also been gained through comparative studies of HTLV-2, a closely related deltaretrovirus that has not been conclusively linked to leukemia or lymphoma [20,21].

In this review, we describe an apoptotic rheostat model in which HTLV-1 rewires the intrinsic and extrinsic apoptotic machinery through the coordinated actions of viral proteins, regulation of pro- and anti-apoptotic BCL-2 family proteins, redox signaling, and immune effector pathways that dynamically tune the balance between leukemic cell survival and apoptosis.

## 2. HTLV-1 Proteins and Cell Turnover

The HTLV-1 RNA genome is about 8500 nt in length and is present in 2 copies in virus particles. After reverse transcription and integration into the host genome, both strands of the double-stranded provirus are transcribed—the plus strand codes for the *gag*, *pro*, *pol*, and *env* genes common to all retroviruses, plus genes coding for regulatory/accessory proteins named Tax, Rex, p21Rex, p30, p13, and p12/p8, and the antisense strand codes for the regulatory protein HBZ [13]. Investigation of the kinetics of HTLV-1 genome expression in individual infected cells showed that plus- and minus-strand transcription occurs with a distinct temporal pattern, with the plus-strand showing defined expression bursts, while minus-strand expression appears to be more constant [22].

The viral gene products Tax and HBZ are the principal drivers of host cell reprogramming culminating in ATLL [23,24]. Indeed, lineage-restricted expression of either protein in transgenic mice is sufficient to induce T-cell malignancies that recapitulate key features of ATLL [25,26].

Tax is a ~40 kDa protein that plays an essential role in viral replication by driving transcription of the ‘sense’ strand of the proviral genome from the 5′ long terminal repeat (LTR) promoter. Tax accomplishes this by forming a complex with the transcription factor CREB and the histone acetyltransferases (HATs) CBP/p300, and PCAF that binds to 3 cyclic AMP response elements (vCREs) in the LTR’s U3 region, resulting in chromatin modifications that permit access by RNA polymerase II [27]. Tax also rewires host transcriptional networks, driving the expression of genes that promote proliferation and survival [28]. Interactions with CREB, AP-1 and SRF contribute to sustained mitogenic signaling [27], whereas activation of the PI3K/AKT [29], mTOR [30] and NF-kB [31,32] pathways promotes cell survival. Tax subverts cell cycle control at multiple levels through functional interactions with E2F transcription factors, cyclin-dependent kinases (CDKs), CDK inhibitors, and the retinoblastoma protein (RB), and impairs DNA damage responses and repair pathways, thereby promoting both proliferation and genomic instability [33]. These combined activities drive the expansion and persistence of infected T-cell clones while increasing mutational burden, ultimately creating a permissive environment for malignant transformation [23].

HBZ is expressed as two nearly identical ~31 kDa isoforms from minus-strand mRNAs transcribed from the 3′LTR [34,35]. Several of the activities described for HBZ oppose those of Tax [24]. HBZ reduces expression of the proviral plus-strand by binding to CREB, which interferes with formation of the Tax/CREB-CBP/p300/PCAF complex at the 5′LTR [36]. HBZ also displaces CREB from cellular CRE, indicating that HBZ can have broad negative effects on CREB-driven gene expression [36]. Additional binding partners for HBZ include the transcription factors ATF, C/EBP, and Smad and Jun family members, and the transcriptional repressor RB. Interactions between HBZ, SMAD3, and p300 enhance TGFβ signaling, which drives expression of FOXP3 [37], a key transcription factor of regulatory T-cells (Treg); this is consistent with a “Treg-like” phenotype described for some ATLL samples showing expression of FOXP3 as well as other canonical Treg-associated surface markers, including CD25, CTLA-4, and HLA-DR [38]. In contrast, Tax can repress TGFβ signaling by interfering with the formation of Smad-containing complexes or with their binding to TGFβ response elements [39].

Activation of the NF-kB pathway plays a crucial role in supporting the proliferation and survival of HTLV-1-infected cells [40], and is affected by both Tax and HBZ. Tax activates both the canonical and non-canonical arms of the NF-kB pathway [41], while HBZ interferes with the activation of the canonical NF-kB pathway [42]. NF-kB activity in HTLV-1-infected cells is also favored by epigenetic silencing of miR-31-5p, whose targets include NIK, an activating component of the non-canonical NF-kB pathway [43]. Analyses of the interplay among Tax, HBZ, and NF-kB indicate that HBZ is needed to maintain the pathway in an optimal state of activation, as unchecked stimulation by Tax would lead to cell senescence [44].

The tumor suppressor protein p53 plays central roles in regulating the response of cells to stress by activating expression of genes that block the cell cycle or promote apoptotic death [45]. Early studies of *TP53* status in the context of ATLL indicated that the gene is not frequently mutated [46], and a more recent next-generation sequencing-based analysis of a large collection of ATLL samples identified *TP53* mutations in 17.8% of the cases [47]. This finding is consistent with the notion that the main mechanism responsible for p53 inactivation in HTLV-1-infected cells is through repression by Tax and HBZ [48,49,50,51]. Tax suppresses *TP53* expression [52] and represses p53 function through antagonistic crosstalk with the NF-kB pathway and sequestration of CREB/ATF and CBP/p300 [48,53,54]. HBZ interferes with p53 function by interfering with its acetylation by CBP/p300 as well as by blocking acetylation of histones at promoters of p53-responsive genes catalyzed by Histone Acetyltransferase Binding to ORC1 (HBO1) [51]. The low frequency of *TP53* mutations observed in ATLL is a property shared with other T-cell malignancies, including T-cell acute lymphoblastic leukemia (T-ALL, derived from immature T-cell precursors) and cutaneous T-cell lymphoma (CTCL, derived from mature CD4^+^ lymphocytes), which frequently show functional inactivation of p53 through CDKN2A/ARF loss, MDM2 activation, defective DNA-damage signaling or deregulated oncogenic kinase and NF-kB pathways [55,56,57].

It is noteworthy that Tax is often silenced in ATLL cells through diverse mechanisms, including DNA methylation, mutations affecting the Tax ORF, and deletions at the 5′ end of the provirus [58,59,60]. Unlike Tax, HBZ expression is maintained in ATLL cells [61], a property that supports HBZ’s role as an essential factor in maintaining the transformed phenotype [62,63]. The silencing of miR-31-5p and consequent deregulation of NIK likely contribute to maintaining NF-kB sufficiently active in ATLL cells that no longer express Tax.

The *HBZ* gene is also functionally relevant independently of its protein-coding capacity. Experiments using an *HBZ* mutant incapable of producing the HBZ protein demonstrated that the HBZ RNA promotes T-cell proliferation via a mechanism involving upregulation of the transcription factor E2F1, a key regulator of the G1-S phase transition [61]. This non-coding activity is consistent with the prominent nuclear accumulation of HBZ transcripts [64]. More recently, the HBZ RNA was shown to participate in silencing of the 5′LTR promoter by interfering with binding of the basal transcription factor TBP [65].

## 3. Apoptotic Control of T-Cell Turnover

Apoptosis is essential for normal development and tissue homeostasis, and its dysregulation of apoptosis contributes to a plethora of diseases, including cancer [66]. Apoptotic death may be initiated by engagement of cell-surface death receptors by their ligands (the extrinsic apoptosis pathway), leading to caspase 8 activation, whereas intracellular stresses engage the intrinsic mitochondrial pathway through BCL-2-family proteins, mitochondrial outer-membrane permeabilization (MOMP), and caspase 9 activation. Both pathways converge on the executioner caspases 3 and 7 [67]. Apoptosis plays a primary role in T-cell homeostasis by eliminating autoreactive T-cells and selectively removing T-cell populations during termination of the immune response [68]. The intrinsic pathway is crucial for all stages of T-cell homeostasis, and the extrinsic pathway comes into play mainly in mature T-cell populations through FAS and TNFR-1 signaling [68,69].

HTLV-1 disrupts several nodes of this network. HTLV-1-infected- and ATLLcells frequently show reduced sensitivity to growth factor withdrawal, genotoxic stress, and death receptor stimulation [70,71,72]. As described below, through Tax and HBZ, HTLV-1 executes a two-branched attack on the apoptotic machinery, increasing the expression/activity of survival proteins that block apoptosis at the mitochondria and downregulating or disabling pro-death factors that would otherwise initiate or execute apoptosis. This coordinated subversion promotes the long-term survival and clonal expansion of infected T-cells, enabling viral persistence and providing a time window for the accumulation of somatic driver mutations that may ultimately lead to ATLL development [73].

## 4. The Intrinsic Apoptotic Pathway and the Critical Role of the BCL-2 Family Proteins

**Figure 1:**
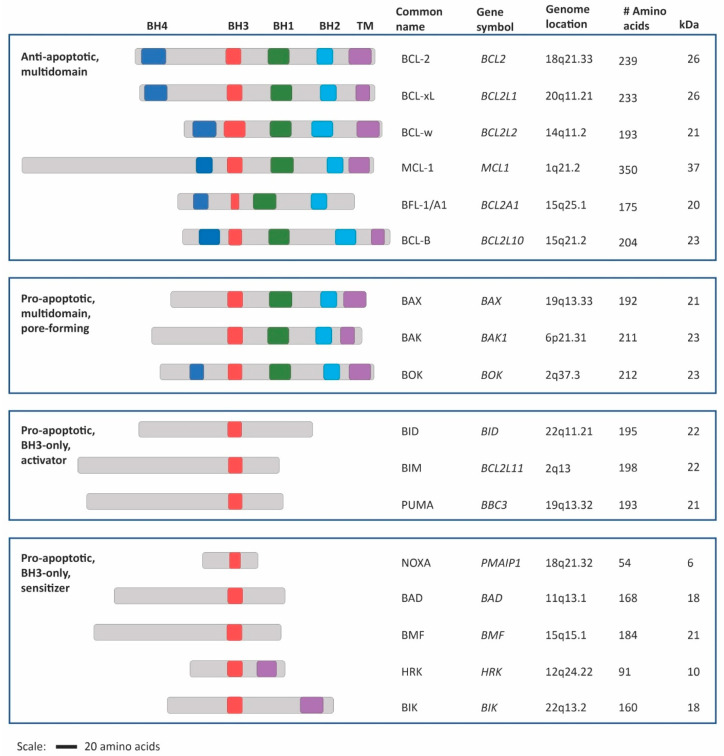
**Anti- and pro-apoptotic BCL-2 family proteins.** Genome location, amino acid number, and calculated mass in kDa are as reported in UniProt (https://www.uniprot.org/, accessed on 10 July 2025). Domain structures of MCL-1, BCL-w, BCL-B, and BFL-1 are from Wu et al. [77], Lindsay et al. [78], Ke et al. [79] and Vogler [80], respectively. Sequence alignments with other BCL-2 family members suggest that BAX and BAK also contain BH4 domains [76]. The activities of the BCL-2 family proteins are governed by protein–protein interactions involving alpha-helical regions in the BH domains. In multidomain proteins, these regions form a hydrophobic groove that confers their ability to associate in homomeric and mixed complexes. The hydrophobic groove of an individual protein can also associate with its own TM domain, preventing the latter from interacting with membranes; this property permits some multidomain proteins to exist in cytoplasmic and membrane-bound forms. The hydrophobic groove also serves as a docking site for BH3-only proteins. Their binding partner specificity identifies 2 categories of BH3-only proteins, the ‘activators’ and the ‘sensitizers’ [81]. The activators (BID, BIM, PUMA) promote apoptosis by binding to pro-apoptotic multidomain proteins, whereas the sensitizers (NOXA, BAD, BMF, HRK, BIK) promote apoptosis when bound to anti-apoptotic proteins. The balance between the levels of pro- and anti-apoptotic BCL-2 family is crucial for determining the cells’ sensitivity to death/survival cues [69].

The intrinsic apoptosis pathway is tightly controlled by the BCL-2 family of proteins, whose archetype, BCL-2, was identified more than 40 years ago [74,75]. The BCL-2 family includes both pro-survival and pro-death members [69] that are distinguished by the presence of BCL-2 homology (BH) domains in two categories: multidomain, which include both pro- and anti-apoptotic members, and BH3-only, all of which are pro-apoptotic (Figure 1). The anti-apoptotic proteins, BCL-2, BCL-xL, BCL-w, MCL-1, BFL-1/A1, and BCL-B, contain all four BH domains and, with the exception of BFL-1/A1, a C-terminal transmembrane (TM) domain. The pro-apoptotic multidomain proteins include BAX, BAK, and BOK, all of which contain BH3, BH1, BH2, and TM domains; BOK also contains a BH4 domain [76].

Intrinsic apoptosis is triggered by specific stress signals, including DNA damage, ER stress, oxidative stress, and growth factor withdrawal. These signals increase the levels of BH3-only pro-apoptotic proteins and/or promote their activation through post-translational modifications and conformational changes, thereby inhibiting anti-apoptotic BCL-2 family members and enabling the pro-apoptotic multidomain proteins to assemble into complexes at the outer mitochondrial membrane. These complexes function as pores that induce MOMP, which permits release of cytochrome *c* and other pro-apoptotic proteins from the mitochondrial intermembrane space into the cytoplasm [82]. Once in the cytoplasm, cytochrome *c* contributes to the formation of the apoptosome, a multimeric complex containing cytochrome *c* and APAF-1 that recruits and activates procaspase 9. Other pro-apoptotic proteins released during MOMP include endonuclease G, which cleaves the nuclear genome, apoptosis-inducing factor (AIF), which is involved in a caspase-independent cell death process termed parthanatos, the serine protease Omi, and Smac/DIABLO, which antagonizes inhibitors of apoptosis proteins (IAP), a family of proteins that interfere with the activity of caspases [83,84].

The anti-apoptotic multidomain BCL-2 family proteins interfere with MOMP by forming complexes with their pro-apoptotic counterparts, thereby blocking pore formation, and by sequestering the activator BH3-only proteins. These activities are counteracted by the occupation of the anti-apoptotic proteins’ hydrophobic groove by sensitizer BH3-only proteins. Understanding of the sensitizer mechanism led to the development of small-molecule drugs termed BH3-mimetics that mimic the function of sensitizers by occupying the hydrophobic groove, thereby inducing apoptotic death of tumor cells that depend on overexpression of anti-apoptotic BCL-2 family proteins for their survival [85,86].

The BH3-only activator protein BID links the extrinsic and intrinsic apoptotic pathways [87]. Under normal conditions, BID is located mainly in the cytoplasm. BID becomes activated upon removal of its N-terminus by specific proteases, including caspase 8, one of the apical caspases of the extrinsic pathway. The resulting activated protein, named tBID, translocates to the outer mitochondrial membrane, where it promotes oligomerization of BAX, BAK, and MOMP, resulting in enhancement of the apoptotic signal [87].

## 5. Reprogramming of Intrinsic Apoptotic Signaling by HTLV-1

HTLV-1-transformed cells exhibit an imbalance in the pro- versus anti-apoptotic proteins of the BCL-2 family. This imbalance is a hallmark of many human leukemias and correlates with therapy resistance [88]. Many of the mechanisms discussed below were identified using HTLV-1-transformed or ATLL-derived cell lines, or ectopic expression systems in cell lines. These models provide important mechanistic insights but may not fully reproduce the biological heterogeneity of primary ATLL. Therefore, mechanisms lacking validation in primary patient samples or independent clinical cohorts should be regarded as model-supported hypotheses rather than universal features of ATLL.

## 6. Effects on Anti-Apoptotic BCL-2 Family Members

**BCL-xL.** Overexpression of BCL-xL is well known to promote multidrug resistance in leukemias [89]. Upregulation of BCL-xL is frequently detected in HTLV-1-infected T-cell lines and ATLL patient samples. High levels of BCL-xL in the context of HTLV-1 were first described in a murine T-cell line that became independent from IL-2 upon stable expression of Tax [90]; the increase in BCL-xL expression was attributed to Tax-mediated effects on NF-ĸB [90], a pathway known to augment BCL-xL expression [91,92]. Subsequent studies showed that BCL-xL is upregulated in cells infected with HTLV-1 or HTLV-2 through the action of their Tax proteins, as well as in ex vivo ATLL samples [93], and that treatment of HTLV-1-transformed cell lines and primary ATLL cells with the NF-kB inhibitor Bay 11-7082 induced downregulation of BCL-xL and triggered apoptosis [94].

**MCL-1.** MCL-1 is distinguished from other BCL-2 family members by its extended N-terminal domain, which comprises nearly half of the 350-amino-acid protein, and by multilayered regulation of its expression, stability and function [95]. The protein has a short half-life due to the presence of N-terminal PEST motifs that are targets of ubiquitination/deubiquitination and phosphorylation that regulate stability and anti-apoptotic activity. These combined post-translational modifications, together with transcriptional, post-transcriptional, and translational control, enable rapid fine-tuning of MCL-1 abundance, and its degradation is a common trigger for apoptosis [95,96].

Proteomic analyses showed that Tax physically interacts with the E3 ubiquitin ligase TRAF6 to catalyze K63-linked non-degradative polyubiquitination of MCL-1. K63-linked ubiquitin chains on MCL-1 prevent its recognition by the 20S proteasome, thereby protecting MCL-1 from degradation under conditions of genotoxic stress. The functional importance of the TRAF6-MCL-1 axis in HTLV-1 infection was demonstrated by (i) shRNA-mediated knockdown of TRAF6 or MCL-1, which triggered apoptosis of both Tax+ and Tax- HTLV-1-infected cell lines, (ii) upregulation of MCL-1 expression in primary human T-cells immortalized by HTLV-1, and (iii) shRNA-mediated knockdown of either MCL-1 or TRAF6, which prevented immortalization of HTLV-1-infected T-cells [97].

HBZ increases MCL-1 stability by interfering with the function of the SCF (Skp1-Cullin1-F-box) ubiquitin ligase complex by binding to Cullin 1 (CUL1) and disrupting its interaction with Skp1, a critical SCF component required for MCL-1 ubiquitination and degradation [98]. This disruption inhibits the proteasomal degradation of MCL-1, causing its accumulation and promoting cell survival. Silencing of HBZ in HTLV-1-infected cells reduces MCL-1 expression, supporting HBZ’s regulatory role in maintaining high MCL-1 levels [98].

The SCF complex is recruited to MCL-1 (and other substrates) through its interaction with FBXW7, a tumor suppressor protein that participates in ubiquitination/proteasomal degradation by binding to proteins phosphorylated by GSK3β. Mutations affecting FBXW7 function have been identified in ATLL cells [99]. FBXW7 can also be functionally inactivated through direct binding to Tax [100].

**Figure 2:**
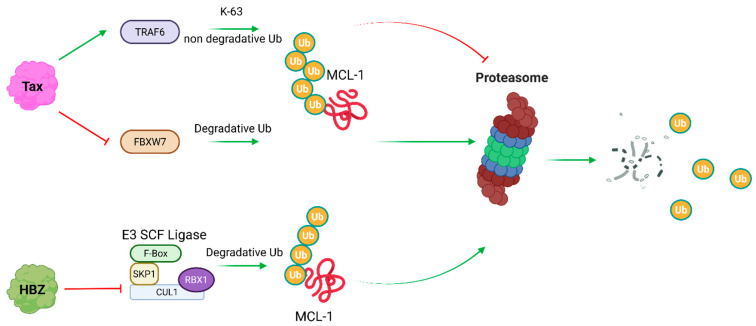
Regulation of MCL-1 ubiquitination and proteasomal degradation in HTLV-1 infection. Tax promotes TRAF6-mediated K63-linked non-degradative ubiquitination of MCL-1 and inhibits the degradative ubiquitination mediated by FBXW7, while HBZ interferes with the SCF complex, stabilizing MCL-1. Green arrows indicate positive regulation of the indicated processes, whereas red T-bars indicate inhibitory effects. Created in BioRender. Darbandi, A. (2026) https://BioRender.com/k9dlc9g (accessed on 8 July 2026).

In addition to its canonical anti-apoptotic function, MCL-1 has been implicated in the regulation of mitochondrial fission/fusion, maintenance of cristae structure, and modulation of autophagic flux under cellular stress, as well as controlling cell cycle progression and senescence [101]. HTLV-1-driven stabilization of MCL-1 is thus likely to exert broad effects, integrating cell turnover with metabolic reprogramming and mitochondrial homeostasis in infected cells. Figure 2 summarizes the effects of Tax and HBZ on MCL-1.

**BFL-1/A1.** Expression of BFL-1 (also known as A1, coded by the *BCL2A1* gene) is a direct transcriptional target of NF-ĸB [102] that is developmentally regulated in T-cells [103]. Macaire et al. found that BFL-1 is expressed in HTLV-1-transformed cell lines and is absent in uninfected T-cell lines [104]. They confirmed BFL-1 expression in 2 primary samples of acute ATLL, while 2 chronic ATLL samples did not express detectable levels of the protein, and showed that transcription of the *BCL2A1* gene is enhanced by Tax via the canonical NF-kB pathway, which synergizes with the AP-1 family transcription factors c-Jun and JunD to maximize *BCL2A1* promoter activation. Interestingly, HBZ was found to modulate this process by inhibiting c-Jun-mediated *BCL2A1* promoter activation, while enhancing JunD’s effect. This suggests that different expression levels of c-Jun or JunD in infected cells could determine their BFL-1 levels. Knockdown of either BFL-1 or BCL-xL induced substantial cell death of HTLV-1-transformed cell lines, while knockdown of BCL-2 had little effect. Furthermore, knockdown of BFL-1 in HTLV-1-transformed cells increased death induced by the BH3 mimetic ABT-737 or the topoisomerase inhibitor etoposide [104].

## 7. Effects on Pro-Apoptotic BCL-2 Family Members

In addition to boosting anti-apoptotic defenses, HTLV-1 dampens pro-apoptotic signals mediated by pore-forming- and BH3-only BCL-2 family proteins.

**BAX.** The pro-apoptotic effector BAX (BCL-2-associated X) is a critical gatekeeper of apoptosis [105,106]. Results of transient transfection assays with reporter plasmids containing the *BAX* promoter in the African green monkey kidney cell line CV-1 indicated that Tax significantly reduced *BAX* promoter activity through a mechanism that involved one of four E-box elements present in the *BAX* 5′UTR [71]. E-boxes are known to serve as docking sites for basic helix-loop-helix (bHLH) transcription factors such as MYC; however, coexpression of MYC did not affect Tax-mediated repression. The study also showed that HTLV-1-transformed and ATLL cell lines were more resistant to apoptosis induced by Taxol, anti-Fas antibody and UV radiation compared to a panel of noninfected T-cell lines; although the authors also reported an association between apoptosis resistance and levels of Tax expression in the HTLV-1-positive cell lines, they did not provide data on Tax expression in the lines [71].

Studies performed on murine fibroblasts showed that Tax blocked serum deprivation-induced apoptosis by preventing the translocation of BAX to mitochondria [107,108]. This inhibition was specific to intrinsic apoptosis, as Tax-expressing fibroblasts remained sensitive to TNFα-induced apoptosis [107]; in this cell system, the effect of Tax on BAX depended on activation of the CREB/ATF-1 transcriptional pathway [108].

Expression of BAX is known to be negatively regulated by microRNAs, including miR-34a-5p [109]. Although miR-34a-5p is downregulated in a variety of cancer histotypes [110], Sharma et al. observed a significant upregulation of miR-34a-5p in HTLV-1-transformed- and ATLL cell lines and a small panel of ATLL samples compared to normal PBMCs [111]. Transfection of an HTLV-1-transformed cell line with a miR-34a-5p mimic resulted in downregulation of BAX mRNA, while transfection with a miR-34a-5p ‘sponge’ induced cell death, suggesting that the microRNA may provide a survival advantage through repression of BAX expression [111].

The studies described above suggest that inhibition of BAX expression by Tax and miR-34a-5p may contribute to the resistance to apoptosis and long-term survival of HTLV-1-infected T-cells, thereby facilitating viral persistence and leukemogenesis. In apparent contrast with these findings, Witzens-Harig and colleagues observed that HTLV-1-transformed- and ATLL cell lines with abundant Tax expression also expressed abundant levels of BAX as well as BCL-2, BCL-xL and BCL-w, whereas lower levels of BAX were detected in a Tax-negative ATLL cell line [112]. Ectopic expression of Tax in the T-ALL cell line Jurkat cells resulted in an increase in levels of BAX as well as BCL-2, BCL-xL, and BCL-w, suggesting a complex role for Tax in modulating the balance of pro- and anti-apoptotic family members [112]. A detailed analysis of expression of the anti- and pro-apoptotic BCL-2 proteins in ATLL samples that are positive and negative for Tax expression, accompanied by experiments in which Tax is transfected or silenced in a more relevant cell system such as ATLL cell lines, would add information to this picture.

**BID and BIM.** BID (BH3-interacting-domain death agonist) and BIM (BCL-2-interacting mediator) are potent BH3-only apoptosis inducers. BID serves as a key link between the extrinsic death receptor pathway and the intrinsic mitochondrial apoptotic pathway. When apoptosis is triggered via death receptors such as Fas or TNFR1, caspase 8 cleaves the full-length BID protein (p22) into its truncated, active form, tBID (p15), thereby initiating its pro-apoptotic activity by inducing oligomerization of BAX or BAK [113] and inhibiting anti-apoptotic BCL-2 proteins, resulting in MOMP. tBID can also directly permeabilize the mitochondrial membrane, independently of BAX and BAK [114]. In T-cells, BIM mRNA expression increases following IL-7 deprivation, suggesting that *BIM* transcription is partially regulated by IL-7 signaling [115]. tBID primarily activates BAK, whereas BIM tends to activate BAX [116].

Immunoblot analyses performed by Mühleisen et al. [117] on a small number of HTLV-1-transformed cell lines, ATLL cell lines, and ATLL patient samples showed that their expression of BIM and BID is markedly reduced compared to control uninfected T-cell lines [117]. Further experiments demonstrated that Tax represses BIM and BID expression through a mechanism involving induction of HIF-1α (hypoxia-inducible factor-1α), which in turn reduces transcription of the *BCL2L11* (encoding BIM) and *BID* genes [118]. Forced expression of BIM or BID in an HTLV-1 T-cell line restored sensitivity to Fas ligand- and TRAIL-induced apoptosis, as well as to chemotherapeutic drugs, suggesting that Tax’s ability to silence BIM and BID is a major contributor to apoptosis resistance [117]. While BID’s role in linking the extrinsic and intrinsic pathways is well known, BIM is typically associated with intrinsic apoptosis. Thus, it seems surprising that BIM alone could sensitize cells to Fas. However, the data suggest that BIM can amplify death receptor signals by lowering the mitochondrial threshold for apoptosis, particularly in cells with suppressed pro-apoptotic signaling. Importantly, pharmacological inhibition of HIF-1α led to an increase in BIM/BID levels and sensitivity to apoptotic stimuli [117]. This Tax-HIF-1α-BIM/BID axis highlights how HTLV-1 suppresses intrinsic apoptosis and promotes anti-apoptotic proteins.

**Figure 3:**
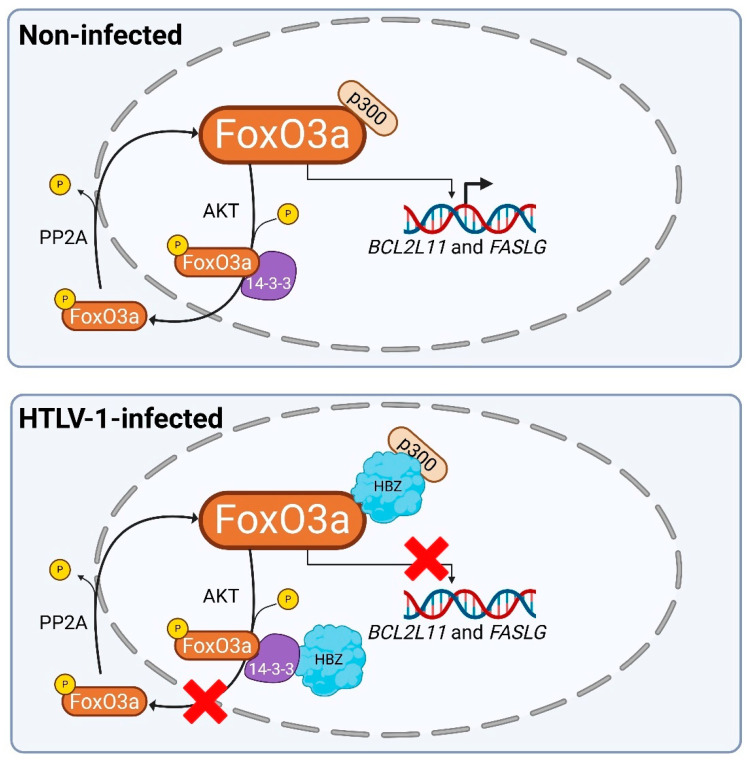
HBZ inhibits FOXO3a-mediated transcription of pro-apoptotic genes. Top panel: in non-infected cells, FOXO3a is dephosphorylated by Protein Phosphatase 2A (PP2A) and translocates to the nucleus, where it associates with transcriptional coactivators such as p300/CBP to induce the expression of BIM and FasL. Phosphorylation of FOXO3a by AKT results in its expulsion from the nucleus in association with 14-3-3 proteins. Bottom panel: in HTLV-1-infected cells, HBZ binds to FOXO3a and p300, disrupting their interaction, thus preventing FOXO3a from binding to DNA and activating its target genes. HBZ also retains the phosphorylated (inactive) form of FOXO3a in the nucleus in a complex with 14-3-3 proteins. Created in BioRender. Darbandi, A. (2026) https://BioRender.com/woedsbd (accessed on 8 July 2026).

HBZ influences BIM expression through an indirect mechanism involving the transcription factor FOXO3a [119]. Tanaka-Nakanishi et al. [119] discovered that HBZ binds to FOXO3a, impairs FOXO3a’s DNA-binding ability and mediates its aberrant retention in the nucleus, resulting in inhibition of the expression of *BCL2L11* and *FASLG* (coding for FasL), 2 of the many pro-apoptotic genes that are regulated by FOXO3a. Details of the HBZ-FOXO3a interaction are shown in Figure 3.

Consistent with these observations, analysis of microarray data indicated downregulation of the BIM and FasL mRNAs in ATLL samples compared to controls, and examination of ATLL cell lines for DNA methylation and histone modifications in the *BCL2L11* promoter indicated epigenetic silencing as an additional possible mechanism repressing BIM expression [119]. By inhibiting FOXO3a-driven expression of BIM and FasL, HBZ helps infected cells evade both intrinsic and extrinsic apoptotic triggers [119,120].

**BAD.** The BH3-only protein BAD (BCL-2-associated agonist of death) plays dual roles as an apoptosis sensitizer and a regulator of cell metabolism. The activity of BAD is determined in large part by the phosphorylation status of serines 75, 99, and 118, which are substrates of diverse kinases, including RSK-1 (Ser75), AKT (Ser99), and PKC iota (all 3 serines) [121]. In its non-phosphorylated form, BAD functions as a sensitizer by associating with BCL-2, BCL-xL, and BCL-w, thereby permitting BAX/BAK oligomerization and MOMP. Phosphorylation of Ser75 and Ser99 suppresses BAD’s pro-apoptotic function by inducing its binding to 14-3-3 scaffold proteins, while phosphorylation of Ser118 interferes with binding of BAD to BCL-2 and BCL-xL [121,122].

AKT-mediated phosphorylation of BAD and consequent suppression of BAD’s apoptotic function [123] is an important contributor to cell survival imposed by the PI3K/AKT pathway, which is known to be activated in HTLV-1-transformed cells and in cells ectopically expressing Tax [29,49,124,125]. Enhancement of PI3K/AKT signaling by Tax results from indirect effects on PTEN and PHLPP, 2 phosphatases that negatively regulate the PI3K/AKT pathway by dephosphorylation of PIP_3_ and AKT, respectively [126]. This effect is mediated by the association of Tax with the PZ-domain protein DLG-1 through a PDZ-binding domain present at the C-terminus of Tax. The Tax-DLG-1 interaction subtracts DLG-1 from PTEN and PHLPP, resulting in the functional inhibition of these phosphatases [126]. The observation that treatment of HTLV-1-transformed cell lines with the PI3K/AKT inhibitor LY294002 led to a decrease in BAD phosphorylation, release of cytochrome c, and activation of caspase 9 provides indirect evidence for a link between Tax-mediated aberrant PI3K/AKT signaling and suppression of intrinsic apoptosis through AKT-mediated phosphorylation of BAD Ser99 and cytosolic sequestration by 14-3-3 proteins [49,123]. Table 1 summarizes the main BCL-2 family proteins modulated by Tax and HBZ.

## 8. Effects on p53-Mediated Apoptotic Signaling

As described in Section 2, the p53 pathway is disrupted in ATLL cells mainly through mechanisms that do not involve *TP53* alterations [48,49,50,51]. p53 plays a central role in regulating apoptosis by upregulating pro-apoptotic proteins and downregulating anti-apoptotic proteins. Multiple components of the intrinsic apoptosis pathway are upregulated at the transcriptional level by p53, including BAX, PUMA, NOXA, BID, BIK, BAD, BIM, and BMF, as well as APAF-1, in most cases through direct binding of p53 to promoter elements [128]. p53 also indirectly represses transcription of the anti-apoptotic proteins BCL-2, BCL-xL, and MCL-1. Furthermore, accumulation of p53 at mitochondria results in its binding to anti-apoptotic BCL-2 proteins [128]. Although mechanistically distinct, both forms of p53 dysfunction (genetic mutation and viral inhibition) may reduce pro-apoptotic signaling and shift the apoptotic balance toward ATLL cell survival.

Another important target of transcriptional repression by p53 is the *BIRC5* gene [129], which codes for Survivin, a member of the IAP (inhibiting apoptosis protein) family of proteins that has multiple activities, including control of mitotic spindle formation and cytokinesis and interference with activation of caspases [130,131]. ATLL cells exhibit upregulation of Survivin [132] and are sensitive to the Survivin inhibitor YM-155 [133]. Activation of the *BIRC5* gene in HTLV-1-infected cells is attributed to Tax-mediated activation of the NF-kB pathway [134] as well as yet-to-be-defined effects of the HBZ RNA [135]; their p53-deficient status also likely contributes to Survivin dysregulation.

## 9. Effects of HTLV-1 on the ROS Rheostat of Cell Fate

Reactive oxygen species (ROS) are homeostatically regulated through the action of feedback loops controlling the balance of ROS-producing electron transfer reactions (e.g., the ETC and several oxidases) and antioxidant systems, e.g., superoxide dismutase (SOD), catalase (CAT), glutathione peroxidase (GPX), glutathione (GSH), and thioredoxin (TRX). It is interesting to note that human TRX was originally identified as ‘ATL-derived Factor’ (ADF), a soluble protein produced by HTLV-1-transformed and ATLL cell lines that induced the expression of the IL-2R [136,137].

By modulating the redox state of key signaling effectors, ROS establishes a tunable “rheostat” that determines the outcome of binary cell fate decisions [138]. At physiological levels, ROS promotes proliferation and pro-survival signaling. In contrast, excessive ROS accumulation induces oxidative damage, driving senescence or apoptosis through pathways such as the DNA damage response, permeability transition pore (PTP) opening, and c-Jun N-terminal kinase (JNK) activation [139].

Cancer cells are characterized by a high ROS setpoint that enhances their fitness by sustaining mitogenic signaling, survival, invasion, metastasis, and angiogenesis. However, this creates a vulnerability, whereby a further increase in ROS levels beyond a toxic threshold engages death pathways [140,141,142,143].

Analyses of PBMCs and serum samples from uninfected controls and HTLV-1-infected individuals provided evidence for a connection between HTLV-1 infection and altered redox homeostasis [144,145,146]. Samples from the infected individuals, comprising healthy carriers and patients with TSP/HAM, showed reduced levels/activity of the antioxidant enzymes CAT, SOD, GPX, glutathione reductase and thioredoxin reductase, a reduction in GSH levels, and increased levels of nitric oxide and malondialdehyde [144,145,146]. Some alterations were more pronounced in patients with TSP/HAM than in asymptomatic carriers, suggesting a possible association with inflammatory disease. To our knowledge, robust data directly comparing basal cytosolic or mitochondrial ROS in purified ATLL cells with normal T-cells and across acute, lymphoma, chronic and smoldering subtypes are currently lacking.

Both Tax and HBZ, as well as the viral accessory protein p13, are involved in regulating redox homeostasis. Ectopic overexpression of Tax in different cell types was shown to induce ROS accumulation, followed by apoptosis [147] or senescence [148]. Tax-induced DNA damage and genomic instability could be blunted by ROS scavengers or by silencing Tax [148]. Tax can also drive transcription of the *TXN* gene (TRX) [149,150], which may contribute to the high levels of TRX (‘ADF’) detected in HTLV-1-transformed and ATLL cell lines [136,137]. Forced expression of TRX in turn represses Tax-driven transactivation of the 5′LTR promoter [149]. This property of TRX, together with the finding that hydrogen peroxide induces Tax-driven transcription from the 5′LTR [149], suggests that the redox-scavenging activity of TRX may promote HTLV-1 ‘latency’, ultimately resulting in increased fitness of infected cells by preventing oxidative stress, excessive viral replication, and immune recognition. A very recent study indicated that Tax-mediated modulation of the ROS rheostat leads to mitochondrial dysfunction and triggers the PINK1-Parkin axis and the NDP52/p62 autophagy receptors, resulting in mitophagy. In this context, mitophagy limits excessive ROS and prevents cGAS-STING activation, thus supporting viral persistence by suppressing type I interferon responses [151].

p13 is targeted to the inner mitochondrial membrane, where it produces an inward K^+^ current that results in increased production of ROS by mitochondria [152]. p13-induced redox modulation was found to exert opposite effects in normal versus leukemic T-cells, promoting activation in the former and triggering apoptosis in the latter due to their elevated oxidative setpoint [153]. These findings suggest that p13 might favor mitotic spread of the virus, while reducing the probability of leukemia development [153,154]. Experiments performed in the T-ALL cell line Jurkat [155] showed that p13 sensitizes the cells to the extrinsic apoptosis stimulus FasL as well as to C2-ceramide, a sphingolipid whose multiple cellular effects include stimulation of intrinsic apoptosis through formation of channels in the outer mitochondrial membrane and enhancement of BAX/BAK oligomerization [156,157]. The relevance of p13 as an apoptotic trigger in the context of HTLV-1-transformation is supported by the finding that siRNA-mediated knockdown of p13 expression reduced cell death in an HTLV-1-transformed cell line [153]. Interestingly, in the presence of Tax, p13 is ubiquitinated and partly targeted to the nucleus, where it binds Tax and inhibits its transcriptional activity [158]. Furthermore, a recent study showed that p13 expression in monocytes/macrophages alters mitochondrial respiration, affects macrophage polarization, and increases CD4^+^ T-cell recruitment [159].

A gene expression analysis of HBZ-expressing HeLa cells versus control cells performed by Rushing et al. suggested a role for HBZ in the antioxidant responses induced by the NRF2 and small Maf (sMaf) transcription factors [160]. The list of oxidative stress-response genes upregulated by HBZ included the gene coding for Heme Oxygenase-1 (*HMOX1*), an enzyme that metabolizes heme groups that are released from heme-containing proteins that have undergone oxidative damage [161]. HMOX-1 expression was found to be elevated in HTLV-1-transformed and ATLL cell lines as well as in CD8^+^-depleted PBMCs from patients with ATLL and TSP/HAM compared to asymptomatic carriers. Dissection of the role of HBZ in inducing HMOX-1 expression revealed that HBZ forms a complex with sMaf transcription factors on a Maf-responsive element (MARE) located in an enhancer upstream of the *HMOX1* promoter; the presence of HBZ in the complex permits recruitment of CBP/p300 to the enhancer, thereby promoting transcription of the *HMOX1* gene [160]. The investigators proposed that this oxidant-protective effect of HBZ may counteract the pro-oxidant effects of Tax and p13 [160]. The data summarized above suggest that Tax, p13 and HBZ may act in concert to reset the ROS rheostat to favor leukemic cell proliferation, survival and chemoresistance, suggesting that targeting the effects of viral proteins on ROS homeostasis could be a promising therapeutic approach, shifting the ROS rheostat toward cell death of transformed cells.

## 10. Resistance to Death Receptor-Mediated Killing

Extrinsic apoptosis is controlled by death ligand-receptor engagement and assembly of the death-inducing signaling complex (DISC) responsible for activation of caspases 10 and 8, the 2 apical caspases of the extrinsic pathway [162]. Many studies have implicated Tax and HBZ in promoting resistance of HTLV-1-infected cells to extrinsic apoptotic stimuli. The first such evidence was obtained in studies demonstrating resistance of HTLV-1-infected cells, Tax-expressing Jurkat cells and lymphocytes from Tax-expressing mice to apoptosis induced by an anti-Fas antibody [163,164], and resistance of infected cell lines to TNF-α [72]. This resistance is conferred in part by Tax-mediated upregulation of XIAP [165] and c-FLIP (cellular FLICE-like inhibitory protein) [127,166] through activation of the NF-kB pathway [165,166]. XIAP is a IAP family member that inactivates caspases 3, 7, and 9 and interferes with mitochondrial release of SMAC/Diablo [167]. c-FLIP is a multitasking survival protein whose role in apoptosis is to compete with binding of pro-caspase 8 to FADD at the DISC (death-inducing signaling complex), resulting in inhibition of caspase 8 activation [168]. In HTLV-1-infected cells, c-FLIP was shown to protect against apoptosis induced by FasL-Fas engagement [127,166]. The increased level of c-FLIP also curbs intrinsic apoptosis by inhibiting caspase 8-mediated cleavage of BID into tBID, which would normally interact with BAX/BAK to amplify the death signal [127,166]. HBZ reinforces the block in FasL-Fas signaling by inhibiting FOXO3a-driven transcription of the *FASLG* gene coding for FasL (Figure 3) [119,120]. Tax-mediated NF-ĸB activation also leads to abundant expression of the ubiquitin-editing enzyme A20 [169], which protects infected cells from apoptosis through a mechanism involving interactions with caspase 8 and FADD [170]. These effects of Tax and HBZ act in the context of a heterogeneous landscape of Fas signaling in ATLL cells. The *FASLG* gene produces alternatively spliced transcripts that code for the membrane-bound Fas protein with receptor function (mFas) and dominant negative soluble Fas isoforms (sFas). Flow cytometry analyses of panels of ATLL samples revealed abundant levels of mFas in most cases [171,172,173] and sensitivity of mFas-expressing ATLL cells to apoptotic death upon exposure to an anti-Fas antibody [171]. Analyses of ATLL samples with loss of mFas expression revealed mutations in the *FASLG* gene that affect splicing and code for Fas variants lacking the transmembrane domain [172,174,175]. Further analyses of the tumor cells and sera from patients with ATLL identified expression of both mFas and sFas in most cases, with patients with acute ATLL expressing higher levels of sFas compared to patients with chronic or smoldering ATLL [173], and high sFas levels associated with clinical markers of poor prognosis [176]. It will therefore be important to correlate the sensitivity of ATLL samples to FasL-induced apoptosis with their levels of membrane-bound and soluble Fas isoforms, Tax and HBZ, and key DISC and caspase-regulatory proteins, together with disease subtype and clinical picture.

## 11. Effects of HTLV-1 on Cytotoxic Lymphocyte-Mediated Apoptosis

The host immune system is equipped with cytotoxic lymphocytes, in particular NK cells and CTL, to recognize and eliminate cells that are harmful to the host, including pathogen-infected cells and cancer cells. CTLs and NK cells can kill target cells either through the surface expression or release of death-receptor ligands such as FasL and TRAIL or the release of perforins and granzymes [177]. Following entry into target cells, granzymes cleave many substrates, including pro-caspase 3, which activates this effector caspase and leads to rapid cell death [178]. Granzyme B promotes apoptosis by cleaving BID into tBID, leading to MOMP, cytochrome c release, and caspase 9 activation [179,180]. Granzyme B also exerts its pro-apoptotic effects in part by activating pro-caspase 3, which degrades MCL-1 [181], and by targeting NDUFV1, NDUFS1, and NDUFS2, core subunits of mitochondrial complex I, leading to impaired activity of the electron transport chain (ETC) and a surge in mitochondrial ROS production. These effects collectively compromise mitochondrial respiration and energy production, contributing to cell death. Similarly, granzyme A (GzmA) induces mitochondrial dysfunction by cleaving components of the ETC, leading to rapid accumulation of ROS and collapse of the mitochondrial membrane potential. The resulting oxidative stress promotes targeting of the SET complex (a key regulator of DNA repair, chromatin remodeling, and apoptosis) to the nucleus [182], where GzmA cleaves SET, relieving its inhibitory effect on nucleases such as NM23-H1, thereby promoting caspase-independent DNA damage and apoptosis [183,184].

HTLV-1 infection is associated with a high frequency of virus-specific CTL, mainly directed against Tax epitopes [185]. Interestingly, the avidity and killing efficiency of HTLV-1-specific CTL were shown to be major determinants of proviral load (PVL) [186], which is an important risk factor for development of ATLL and TSP/HAM [187]. A search for correlations between CD8^+^ T-cell numbers/function and PVL revealed increased expression of GzmA, perforin 1 and NKG2B in CD8^+^ cells isolated from infected carriers with low PVL compared to those with high PVL and in carriers with low PVL compared to uninfected controls [188]. However, elevated expression of anti-apoptotic proteins of the BCL-2 family in ATLL cells may counteract the pro-apoptotic effects by granzymes [189]. A potential consequence is the generation of “frustrated” CTLs that repeatedly engage resistant target cells without achieving cytolysis, thereby contributing to T-cell exhaustion and promoting immune escape [190,191].

Furthermore, Tax-specific CTL from patients with ATLL are less abundant and recognize fewer epitopes compared to those found in asymptomatic carriers [192]. Overcoming these defects in CTL response may thus be effective in controlling ATLL [193].

## 12. Therapeutic Targeting of BCL-2 Family Proteins in ATLL

Given the central role of BCL-2 family proteins in ATLL cell survival, there has been great interest in targeting these molecules therapeutically to tip the balance back toward apoptosis in ATLL cells. Several strategies have emerged.

## 13. BH3 Mimetics

BH3 mimetics are small molecules that bind to the hydrophobic groove of the anti-apoptotic BCL-2 family proteins, resulting in BAX/BAK oligomerization, MOMP, cytochrome c release and commitment to apoptosis.

In 2005, Oltersdorf and colleagues described ABT-737, a small molecule that selectively binds to the anti-apoptotic BCL-2 family proteins, including BCL-2, BCL-xL, and BCL-w, with high affinity and induces BAX/BAK-dependent apoptosis [194]. This groundbreaking discovery laid the foundation for the development of the clinically approved BH3-mimetic Venetoclax, as well as many other compounds under clinical testing [88].

Ishitsuka et al. reported that ABT-737 induces extensive apoptosis in HTLV-1-infected T-cell lines and in primary ATLL cells ex vivo [195]. Treated ATLL cells showed activation of caspases and cell death, indicating that they are heavily dependent on BCL-2 and/or BCL-xL for survival. Moreover, ABT-737 significantly enhanced the cytotoxicity of conventional chemotherapeutic drugs when used in combination [195]. In a mouse model of aggressive ATLL, ABT-737 markedly inhibited tumor growth in vivo, providing proof of concept that targeting BCL-2 family proteins can overcome ATLL’s drug resistance and that BH3 mimetics might represent a promising therapeutic approach, either alone or in combination with other drugs [195].

Navitoclax (ABT-263) is an orally bioavailable derivative of ABT-737 that was first described in 2008 [196]. Although effective in killing neoplastic cells, Navitoclax is still under clinical evaluation due to severe thrombocytopenia [197] caused by inhibition of BCL-xL, which is an essential survival factor for platelets [198]. Witzens-Harig et al. demonstrated that Navitoclax induced efficient killing in a small panel of ATLL cell lines and HTLV-1-transformed cell lines, showing 10 to 20-fold lower EC50 values compared to HTLV-1-negative T-cell lines [112]. This heightened sensitivity correlated with the cells’ elevated expression of BCL-2, BCL-xL, and BCL-w, as well as BAX and Tax. Interestingly, the cells became less sensitive to the drug upon BAX knockdown, confirming the central role of BAX as a determinant of response [112]. Further experiments in Jurkat cells showed that Tax upregulated BAX and increased the cells’ sensitivity to Navitoclax, suggesting that Tax-mediated BAX upregulation may prime cells for death induced by BH3 mimetics. The findings by Witzens-Harig et al. raised the prospect that low-dose Navitoclax may have clinical benefit in ATLL, or that BH3 mimetics could be used as chemosensitizers to lower doses of conventional agents [112].

Venetoclax (ABT-199) is an orally bioavailable selective BCL-2 inhibitor developed to induce apoptosis in cancer cells overexpressing BCL-2 [199] without harming BCL-xL-dependent platelets. Venetoclax is approved by both the US Food and Drug Administration (FDA) and European Medicines Agency (EMA) for the treatment of chronic lymphocytic leukemia (CLL) [200] and Acute Myeloid Leukemia (AML) [201,202].

In ATLL, where BCL-xL and other anti-apoptotic BCL-2 family proteins play a major role, single-agent Venetoclax may be less effective. However, a recent case report by Petrillo et al. described two patients with relapsed/refractory ATLL (one with a lymphoma and the other with a leukemic presentation consistent with acute ATLL) who achieved complete remission in response to Venetoclax, accompanied by a significant reduction in HTLV-1 proviral load. Both patients subsequently underwent successful allogeneic stem-cell transplants [203]. In the absence of functional analyses to define apoptotic dependencies, it remains unclear whether these tumors were truly BCL-2-dependent.

The fact MCL-1 and BFL-1 are upregulated by both Tax and HBZ suggests that MCL-1/BFL-1 inhibitors might be particularly effective for killing ATLL cells (which often express only HBZ) as well as infected cells at earlier stages of transformation (which express both Tax and HBZ). While BFL-1 inhibitors are still at the in vitro preclinical testing phase [204], faster progress has been made for MCL-1 inhibitors, including MIK665 and AMG-176, which have undergone initial clinical testing for treatment of AML, multiple myeloma and other malignancies [205]. S63845, a parent molecule of MIK665, was recently tested in a study that investigated the biological/pathological properties of humanized mice infected with HTLV-1 subtype C, a molecular subtype that is associated with aggressive disease in an infected Aboriginal population of Australia [206]. The interesting findings in this study included the observation that the CD4^+^ cells isolated from the HTLV-1c-infected mice showed increased expression of MCL-1 compared to cells from uninfected mice, and that S63845 induced apoptosis of the infected cells ex vivo and reduced expansion of the infected CD4^+^ cell population in vivo, thus suggesting a dependency on MCL-1 in this context [207].

Taken together, these observations suggest that combined targeting of multiple anti-apoptotic proteins, including BCL-xL, MCL-1, and BFL-1, may represent a rational strategy to overcome the multilayered anti-apoptotic network in ATLL.

## 14. Combining BH3 Mimetics with Other Therapies

Because ATLL cells often co-express multiple anti-apoptotic proteins, e.g., MCL-1 and BFL-1, which are not targeted by Navitoclax or Venetoclax, combination strategies are being explored to achieve broader induction of apoptosis.

**Proteasome Inhibition.** Proteasome inhibition impacts several survival pathways, including NF-kB and BCL-2 family proteins, and p53 [208]. Bortezomib (PS-341), a clinically approved proteasome inhibitor, was shown to induce growth inhibition, cell cycle arrest and apoptosis in HTLV-1-transformed and ATLL cell lines [209], and displayed antitumor activity when combined with the anti-IL-2R monoclonal antibody Zanapax in an ATLL xenograft mouse model [210]. The effects of Bortezomib were attributed to inhibition of NF-kB signaling through stabilization of IkB protein [209,210] as well as an increase in the levels of p21, p27, and p53 [209]. Interestingly, ceramide accumulation was observed in the Bortezomib-treated cells and correlated with apoptosis; however, the drug also induced Tax stabilization, which may blunt the drug’s effects on NF-kB signaling [209].

Based on these preclinical findings, a multi-center phase II clinical trial was conducted to assess the efficacy of Bortezomib in patients with relapsed or refractory ATLL (Clinical Trial Registry: UMIN000004061) [211]. Among 15 patients enrolled in the first stage of the study, one underwent a partial remission and five achieved stable disease; however, the study was terminated due to the modest benefit provided by the single agent therapy [211].

Kunami et al. tested ABT-737 in combination with Bortezomib in HTLV-1-transformed and ATLL cell lines and freshly isolated ATLL cells [212]. Results showed that this drug combination significantly enhanced apoptosis in the cell lines and ATLL samples, demonstrating a synergistic effect. Bortezomib increased the expression of both NOXA and MCL-1, without significantly altering levels of BCL-2, BCL-w, or BCL-xL. Immunoprecipitation assays revealed enhanced formation of the MCL-1-NOXA complex following treatment with either Bortezomib alone or the ABT-737/Bortezomib combination. In this setting, the upregulation of NOXA functionally inactivated MCL-1, thereby sensitizing cells to ABT-737, which, as stated earlier, targets BCL-2, BCL-xL, and BCL-w [212].

**Histone Deacetylase Inhibitors (HDACi).** In hematological malignancies, HDACi were shown to modulate the expression of BCL-2 family proteins by downregulating BCL-2, BCL-xL, and MCL-1, while upregulating BIM, BID, BAK, and BAX, thereby shifting the balance toward apoptosis in cancer cells [213,214,215,216]. Several HDAC inhibitors, such as Panobinostat, Vorinostat (SAHA), Romidepsin, Belinostat, and Chidamide, have demonstrated efficacy in preclinical studies and clinical trials involving ATLL cell lines, primary ATLL cells, and patients with relapsed or refractory disease [217,218]. The above-mentioned study by Kunami et al. also showed that ABT-737 synergistically induced apoptosis in combination with SAHA in HTLV-1-transformed- and ATLL cell lines and ATLL samples [212]. This observation is supported by studies of a MYC-driven lymphoma model showing that overexpression of BCL-2 or BCL-xL reduced the effectiveness of HDAC inhibitors [219].

**JAK1/2 Inhibition.** HTLV-1-transformed cells show constitutive activation of the JAK/STAT pathway, a property that correlates with the cells’ acquisition of IL-2 independence [220]. A pivotal study by Zhang et al. demonstrated that the selective JAK1/2 inhibitor Ruxolitinib synergizes with the BCL-2/BCL-xL/BCL-w inhibitor Navitoclax to induce apoptosis in ATLL models [221]. The rationale for this dual targeting stems from HTLV-1 Tax-mediated activation of the multiple common γ-chain cytokine pathways (including IL-2, IL-15, and IL-9), thus driving persistent JAK/STAT signaling. In IL-2-dependent ATLL cell lines, co-treatment with Ruxolitinib and Navitoclax resulted in MOMP, caspase 3/7 activation, and cleavage of MCL-1 to a 24 kDa pro-apoptotic isoform [221]. Notably, the combination elevated active BAX levels and reduced mitochondrial membrane potential, both of which are key features of intrinsic apoptosis. The combination also enhanced apoptosis ex vivo in smoldering/chronic ATLL patient-derived PBMCs and in a murine xenograft model. Mechanistically, JAK inhibition led to reduced p-STAT5, upregulation of pro-apoptotic BH3-only proteins such as BIM and PUMA, and reduced p-BAD, thereby priming the cells for Navitoclax-induced mitochondrial apoptosis. The cleaved form of MCL-1 observed in co-treated cells further supports the idea that BH3 mimetics can convert MCL-1 into a pro-death factor under certain conditions. These findings suggest that dual targeting of JAK/STAT signaling and BCL-xL may overcome apoptotic resistance, particularly in BCL-xL-dependent ATLL cells. This combinatorial strategy holds translational promise for clinical application, especially in smoldering and chronic ATLL subtypes characterized by IL-2 dependence and active JAK/STAT signaling [221].

**Immunotherapy and Apoptosis.** Emerging immunotherapeutic strategies, such as the anti-CCR4 monoclonal antibody mogamulizumab, mediate the depletion of ATLL cells primarily through antibody-dependent cellular cytotoxicity (ADCC) [222]. However, it is plausible that tumor cells resistant to granzyme-induced apoptosis may exhibit reduced susceptibility to ADCC-mediated cytotoxicity. In this context, combining immunotherapy with BH3 mimetics may offer a rational strategy for enhancing therapeutic efficacy by lowering the apoptotic threshold and facilitating execution of cell death downstream of cytotoxic immune signals. This concept is supported by recent studies in T-ALL, where BH3 mimetics enhanced NK cell-mediated killing and induced additive or synergistic anti-leukemia effects in cell lines and patient-derived xenograft samples [223]. Although the relevance of these findings to ATLL remains to be established, they provide a rationale for future preclinical evaluation of combined immunotherapy and apoptosis-targeting approaches in ATLL. Table 2 summarizes the inhibitors and combination strategies evaluated in HTLV-1 infection and ATLL, together with the experimental models or patient populations studied, the main findings, and the limitations of the available evidence.

## 15. Conclusions and Future Perspectives

HTLV-1-driven leukemogenesis is tightly linked to a progressive rewiring of the intrinsic apoptotic machinery, in which viral proteins reshape the BCL-2 family network through complementary mechanisms to upregulate anti-apoptotic proteins such as BCL-xL, MCL-1, and BFL-1, while suppressing or functionally disabling pro-apoptotic effectors, including BAX, BIM, BID, BAD, FasL and caspases. This survival-biased rheostat not only protects infected cells from intrinsic stress signals but also blunts immune-mediated killing and contributes to the profound therapy resistance that characterizes ATLL.

Notably, the effects of Tax are likely to be highly context-dependent, as results obtained in monkey kidney cells [71] and mouse fibroblasts [107,108] suggest a negative regulation by Tax, while transfection of Tax in the Jurkat cell line shows an opposite effect [112]. Future studies should address this point by testing these mechanistic connections with loss-of-function approaches in more HTLV-1-relevant cell contexts, e.g., primary mature T-cells and infected cells.

The mechanistic insights resulting from studies of the BCL-2 family identify mitochondrial apoptosis as a potential actionable vulnerability in HTLV-1-associated malignancy. Preclinical and emerging clinical evidence supports the use of BH3 mimetics, particularly in rational combinations with proteasome inhibitors, HDAC inhibitors, JAK/STAT inhibitors, and possibly antiretroviral therapy to overcome survival pathways and restore apoptotic competence. Redox-directed strategies should also be viewed as mechanistically connected to BCL-2 targeting, as indicated by studies in T-ALL that showed that increasing mitochondrial ROS can selectively kill leukemic cells [224,225], and that mTOR inhibition can impinge on ROS homeostasis [226] and sensitize T-ALL cells to Venetoclax through sustained engagement of the integrated stress response [227]. These findings derived from T-ALL have not yet been directly established in HTLV-1-associated ATLL; therefore, their relevance to ATLL should currently be regarded as a testable hypothesis rather than a demonstrated therapeutic mechanism.

Extending this scenario to ATLL, it would be interesting to test whether ROS elevation may sensitize leukemia cells to BH3 mimetics. However, the marked heterogeneity of ATLL suggests that no single anti-apoptotic dependency will apply universally. Future efforts should therefore prioritize functional BH3 profiling, integrated genomic and transcriptomic analyses, and biomarker-guided patient stratification to define the dominant survival circuitry in individual tumors. In parallel, further investigation of how viral latency, microenvironmental cues, and immune escape intersect with BCL-2 signaling may reveal additional therapeutic opportunities.

In summary, framing HTLV-1-driven leukemogenesis through its impact on the apoptotic rheostat provides a mechanistic rationale for combining BH3 mimetics, redox-modulating agents, and immune-directed therapies in patients with ATLL.

## Figures and Tables

**Table 1:** HTLV-1-mediated modulation of apoptosis-related proteins.

Protein	Pro-/Anti-Apoptotic	Viral Modulator	Mechanism of Modulation	Experimental Model(s)	Reference s
BCL-xL	Anti-apoptotic	Tax	Transcriptional activation via NF-kB	HTLV-1 +/− T-cell lines, primary ATLL cells (*n* = 6), Jurkat Tax+/−, Murine Tax + T-cell line	[90,93,94]
MCL-1	Anti-apoptotic	Tax, HBZ	Post-translational stabilization via TRAF6 (Tax); reduced ubiquitination (HBZ)	Jurkat Tax or HBZ+/−, HTLV-1 + T-cell lines, non-T-cell lines, normal PBMC infected with HTLV-1 and depleted for MCL1 or TRAF6, non-T-cell lines	[97,98]
BFL-1/A1	Anti-apoptotic	Tax, HBZ	Transcriptional activation via NF-kB/AP-1	HTLV-1 +/− T-cell lines, non-T-cell lines, primary ATLL cells (*n* = 4; 2 acute, 2 chronic)	[102,104]
BCL-w	Anti-apoptotic	Tax	Not well defined	Jurkat Tax+/−, HTLV-1 +/− T-cell lines	[112]
BAX	Pro-apoptotic	Tax	Transcriptional repression via E-box binding; functional inhibition by anti-apoptotic BCL-2 family proteins	CV-1 Tax+/−, HTLV-1 +/− T-cell lines, murine fibroblast cell line (Tax+/−)	[71,107,108,112]
BID	Pro-apoptotic	Tax	Transcriptional downregulation via HIF-1α activation	HTLV-1 +/− T-cell lines, HTLV-1-infected patients (*n* = 5), Jurkat and Hela Tax+/−, Jurkat si BIM and BID, HTLV-1 (+) si HIF-1α	[117]
BIM	Pro-apoptotic	Tax, HBZ	Transcriptional downregulation via HIF-1α (Tax); inhibition of transcription via FOXO3a (HBZ)	HTLV-1 +/− T-cell lines, HTLV-1 infected patients (*n* = 5), Jurkat and Hela Tax+/−, Jurkat si BIM and BID, T-ALL cell lines Jurkat and CEM HBZ+/−, HTLV-1 (+) si HIF-1α, primary ATL cells (*n* = 7), microarray dataset (GSE33615)	[117,119]
BAD	Pro-apoptotic	Tax (potentially)	Inactivation via PI3K/Akt-mediated phosphorylation	Not experimentally tested	[123]
FasL	Pro-apoptotic	HBZ	Transcriptional downregulation via FOXO3a inhibition	Jurkat and CEM HBZ+/−, HTLV-1 +/− T-cell lines, microarray dataset (GSE33615)	[119]
c-FLIP	Anti-apoptotic	Tax	Transcriptional upregulation via NF-kB; inhibits CD95/Fas-mediated caspase 8 activation	Jurkat Tax +/− (transfected with c-FLIP siRNA), HTLV-1 +/− T-cell lines	[127]

**Table 2:** Preclinical and clinical evidence for apoptosis-targeted therapies in HTLV-1 infection and ATLL.

Inhibitor or Combination	Experimental Model or Study Population	Interpretation and Limitations	Refs.
ABT-737	HTLV-1-infected- and ATLL-derived T-cell lines; primary acute ATLL samples; mouse xenograft model.	Provides preclinical proof of concept for targeting BCL-2-family dependencies in ATLL; clinical efficacy has not been established.	[195]
Navitoclax (ABT-263)	ATLL- and HTLV-1-transformed T-cell lines; Jurkat cells expressing Tax	Findings are based on cell lines.	[112]
Venetoclax (ABT-199)	Two patients with relapsed or refractory ATLL	Evidence is limited to two cases.	[203]
S63845	CD4^+^ cells infected with HTLV-1 subtype C; humanized mouse model of HTLV-1 infection	Supports an MCL-1 dependency in the HTLV-1 subtype C model; confirmation in primary human ATLL samples is required; clinical efficacy has not been established.	[207]
Bortezomib	ATLL- and HTLV-1-transformed T-cell lines; Fifteen patients with relapsed or refractory ATLL enrolled in the first stage of a multicenter phase II trial	The trial was terminated because single-agent treatment provided only modest clinical benefit.	[210,211]
ABT-737 + Bortezomib	ATLL- and HTLV-1-transformed T-cell lines; freshly isolated acute ATLL cells	Promising preclinical combination, but clinical efficacy has not been established.	[212]
ABT-737 + SAHA/Vorinostat	ATLL- and HTLV-1-transformed T-cell lines; freshly isolated acute ATLL cells	Promising preclinical combination, but clinical efficacy has not been established.	[212]
Navitoclax + Ruxolitinib	IL-2-dependent ATLL cell lines; PBMCs from patients with smoldering or chronic ATLL; cell-line-derived mouse xenograft model	Provides strong preclinical rationale, particularly for smoldering or chronic ATLL; clinical benefit remains unconfirmed.	[221]
Immunotherapy + BH3 mimetics	Supporting studies conducted in T-ALL cell lines and patient-derived xenograft samples	This is a proposed future direction. Its relevance to ATLL has not yet been established experimentally.	[223]

## Data Availability

No new data were created or analyzed in this study.
